# Attention to fat- and thin-related words in body-satisfied and body-dissatisfied women before and after thin model priming

**DOI:** 10.1371/journal.pone.0192914

**Published:** 2018-02-15

**Authors:** Leah N. Tobin, Christopher R. Sears, Alicia S. Zumbusch, Kristin M. von Ranson

**Affiliations:** Department of Psychology, University of Calgary, Calgary, Alberta, Canada; Northeastern University, UNITED STATES

## Abstract

Understanding the cognitive processes underlying body dissatisfaction provides important information on the development and perpetuation of eating pathology. Previous research suggests that body-dissatisfied women process weight-related information differently than body-satisfied women, but the precise nature of these processing differences is not yet understood. In this study, eye-gaze tracking was used to measure attention to weight-related words in body-dissatisfied (*n* = 40) and body-satisfied (*n* = 38) women, before and after exposure to images of thin fashion models. Participants viewed 8-second displays containing fat-related, thin-related, and neutral words while their eye fixations were tracked and recorded. Based on previous research and theory, we predicted that body-dissatisfied women would attend to fat-related words more than body-satisfied women and would attend to thin-related words less. It was also predicted that exposure to thin model images would increase self-rated body dissatisfaction and heighten group differences in attention. The results indicated that body-dissatisfied women attended to both fat- and thin-related words more than body-satisfied women and that exposure to thin models did not increase this effect. Implications for cognitive models of eating disorders are discussed.

## Introduction

Dissatisfaction with one’s body shape or size is common among women in Western cultures [1]. For example, as many as 72% of U.S. women report some degree of body dissatisfaction [2]. Dissatisfaction with one’s body is linked to poor peer relationships [3], as well as substance use, early sexual activity, self-harming behaviours, and suicidal ideation [4, 5]. Research also suggests that body dissatisfaction is a causal risk factor for the development of eating disturbances in adolescent girls and women [6, 7]. Given these negative outcomes, it is important to understand the factors influencing the development and maintenance of body dissatisfaction, as this knowledge can inform treatment and prevention efforts.

### Attentional biases and the cognitive model of eating disorders

A great deal of research has examined cognitive biases (i.e., attention, memory, and interpretation biases) in individuals with body dissatisfaction and eating pathology (e.g., [8–13]). Existing reviews of attentional biases among women with eating disorders [14, 15] indicate that in experimental studies women with eating disorders exhibit a consistent attentional bias for disorder-relevant stimuli (i.e., body- or food-related stimuli). Theoretical models often posit that attentional biases play a role in the development and maintenance of eating disorders, though a comprehensive account of the role attentional biases play in eating disorders has not yet been developed [14]. One of the proposed models, Vitousek and Hollon’s [16] cognitive model of eating disorders, has provided the dominant theoretical framework to account for the existence and nature of attentional biases in those with eating pathology. According to this model, individuals with eating pathology develop self-schemas (a set of beliefs and generalizations about themselves) that focus on weight and negative thoughts about weight and other body-related attributes (e.g., size, shape). The schemas are believed to influence an individual’s thoughts, affect, and behaviour. Maladaptive schemas are proposed to constitute the core cognitive component of eating disorders and to lead to selective processing of information related to weight, which can manifest as biased attention in individuals with eating pathology.

Researchers who have examined attentional biases in individuals with eating pathology and body dissatisfaction have interpreted Vitousek and Hollon’s [16] model in different ways, which has led to different predictions for how attentional biases should manifest. Some researchers have interpreted the model to predict that maladaptive schemas in those with eating pathology or body image difficulties will lead to preferential processing of schema-congruent (e.g., “fat-related”) information and avoidance of schema-incongruent (e.g., “thin-related”) information [9, 11]. According to this interpretation, individuals with eating pathology direct their attention toward perceived flaws in their body shape or weight while ignoring positive features of their appearance [17–19], and for some this propensity extends to their perception of other individuals. Gao et al. [11] found empirical support for this prediction. Using a dot-probe task, weight-dissatisfied and weight-satisfied women responded to visual probes appearing in the location of fat-related, thin-related, and neutral words. The word pairs were presented for 1000 ms before the probe appeared, and eye gaze was monitored during the dot-probe task. Gao et al.’s analyses revealed an orientation bias, such that weight-dissatisfied women had more frequent initial fixations on fat-related words than weight-satisfied women (for thin-related words there was no group difference). Gao et al. also found that weight-dissatisfied women had shorter gaze durations to thin-related words than weight-satisfied women, whereas for fat-related words there was no group difference. Their results support the prediction that individuals with negative self-schemas related to weight exhibit attentional biases for fat-related information (more frequent initial fixations to fat-related words compared to weight-satisfied women) and an avoidance of thin-related information (shorter gaze durations for thin-related words compared to weight-satisfied women). Several other studies have also found biases in attention that suggest that body-dissatisfied women [20] and women with an eating disorder [21, 22] exhibit heightened attention to fat-related images or words and either avoid or exhibit no bias for thin-related information.

An alternative interpretation of Vitousek and Hollon’s [16] model is that maladaptive schemas in body-dissatisfied women lead to preferential processing of all weight-related information (both “negative” fat-related information and “positive” thin-related information). This interpretation of the model has been supported by several studies. These studies have found that women with an eating disorder [10, 23, 24] and women with high levels of body-dissatisfaction [12, 13] exhibit heightened attention to both weight- and body-related words, including fat-related and thin-related words. For example, in another study, Gao et al. [12] showed women who were high or low in body dissatisfaction displays of four images, each display consisting of one “fat” body image or one “thin” body image, and three control images. The four images were presented for 15 seconds, during which time participants’ eye gaze was tracked and their fixations to the “fat” and “thin” body images were measured. Gao et al. found that women high in body dissatisfaction had longer fixation times for both “thin” and “fat” body images relative to women low in body dissatisfaction during the entire 15-second presentation time. Their results provide empirical support for an interpretation of Vitousek and Hollon’s model that leads to the prediction that women with negative self-schemas related to weight will exhibit similar attentional biases for both fat- and thin-related information.

The purpose of the present study was to clarify how attentional biases manifest in body-dissatisfied women, given these mixed findings in the literature. As described above, both interpretations of Vitousek and Hollon’s model [16] predict that body-dissatisfied women will exhibit biased attentional processing of weight- and body-related information, but they differ with respect to the specificity of the bias. Our study was designed to distinguish between: 1) a specific attentional bias for fat-related information, coupled with an attentional avoidance of thin-related information, which would manifest as heightened attention to fat-related information and reduced attention to thin-related information relative to body-satisfied women, and 2) a general bias in attention to weight-related stimuli, which would manifest as heightened attention to both types of weight-related stimuli (fat- and thin-related) relative to body-satisfied women. Distinguishing between these two possibilities is important because an understanding of biased attention in body-dissatisfied women informs the content and efficacy of prevention efforts, including attentional bias modification treatments designed to train individuals to direct their attention to or away from different treatment targets [25]. Identifying specific treatment targets for this at-risk population will be valuable, because the biases are considered mechanisms that influence the maintenance of psychological processes related to eating disorders (e.g., by perpetuating maladaptive schemas through selective processing of schema-congruent information).

### Priming attentional biases in women at risk for an eating disorder

For researchers studying eating disorders, an understanding of attentional biases in non-clinical samples is important given that it could help identify those at elevated risk for development of an eating disorder. One advantage of studying at-risk, non-clinical samples is that potential confounding effects of comorbid psychopathology (e.g., affective or anxiety disorders) or functional impairment are less likely to be present [11]. To date, empirical support for Vitousek and Hollon’s [16] model in non-clinical populations has been mixed. In a meta-analytic review [10], Dobson and Dozois concluded that there was no definitive evidence of attentional biases in non-clinical, at-risk (i.e., body-dissatisfied, dieting, or food-restricting) samples of women, whereas there was clear evidence of attentional biases for weight-related words among women with eating disorder diagnoses. However, several recent studies that used a variety of different methods (e.g., eye-gaze tracking, the dot-probe task, and the dichotic listening task) have found evidence of attentional biases in non-clinical at-risk samples [11, 12, 26].

One interpretation of the mixed findings with non-clinical samples is that those at risk for developing an eating disorder have attentional biases that are latent and less readily observable than the biases in clinical samples. For example, another meta-analysis of studies examining attentional biases for food- and body-related words [15] obtained results similar to those reported by Dobson and Dozois [10], but concluded that the small effect size for non-clinical samples did not necessarily indicate an absence of an effect. They also concluded that it is possible that activation of relevant concerns is required to observe larger attentional biases in non-clinical individuals. Researchers have theorized that viewing thin model images may activate (or prime) an individual’s maladaptive body image schema through social comparison and self-discrepancy, given that mass media images of fashion and fitness models have been demonstrated to increase women’s self-reported body dissatisfaction [27, 28].

Although thin model exposure has been shown to increase self-reported body dissatisfaction, empirical support for the proposal that thin model exposure can influence attentional biases has been equivocal [9, 13, 29, 30]. Markis and McLennan [13], for example, sought to activate women’s maladaptive body image schemas by showing images of thin models. They then measured attentional biases to body-related words using a Stroop task [31], a response latency-based measure of attentional engagement. They reported a correlation between self-reported body dissatisfaction and attentional biases to body-related words, such that higher levels of self-reported body dissatisfaction were associated with slower response times to body-related words in the Stroop task (note that they did not distinguish between body-related words that were fat- or thin-related). Cassin et al. [9], on the other hand, found that there was no effect of thin model exposure on attentional biases in women at risk for development of an eating disorder. One complication when interpreting this literature is that different studies have used different priming procedures and methods for measuring attentional bias, and so it is difficult to determine if the inconsistent findings are due to a specific priming procedure or the limitations of a particular attention task.

### The present study: Aims and hypotheses

The purpose of the present study was to ascertain the nature of attentional biases related to women’s body dissatisfaction and to clarify whether exposure to images of thin models would make these biases more salient by priming participants’ negative weight-related self-schemas. We used eye-gaze tracking to measure attention to both fat- and thin-related words over an 8-second interval in women with high versus low body dissatisfaction, before and after the women experienced a priming condition that involved viewing thin model images. Body dissatisfaction was measured before and after exposure to the thin model prime (i.e., immediately after each eye-tracking assessment) to assess the efficacy of the priming procedure. Whereas both words and images have been used as stimuli to measure attention to weight-related information (e.g., [10–12]), images may trigger upward or downward appearance comparisons [12] that could influence attention. To avoid this complication, we used fat- and thin-related words to measure attentional biases associated with body dissatisfaction, because with words it is less likely that appearance comparisons will affect attention. Words also allow for precise control of a wide variety of features (e.g., number of letters, normative frequency, valence), which increases the internal validity of a study, whereas with images this level of control is much more difficult to achieve. Several studies have used eye-tracking methodology to measure attention to threat-related words in posttraumatic stress disorder (e.g., [32]) and to positively- and negatively-valenced words in dysphoric and depressed individuals (e.g., [33]).

An eye-tracking paradigm has significant advantages over response latency-based measurements of attention that use speeded responses to stimuli to infer where a participant is attending at a single moment in time (e.g., the dot-probe task). Eye-gaze tracking can provide a direct and continuous measure of a participant’s attention by measuring individual fixations to stimuli as they are attended to and examined over time. The ability to measure shifts of attention over extended intervals allows researchers to distinguish between early and late phases of attentional engagement with stimuli, which is especially valuable when there are competing stimuli in a display [32, 34]. For our study, we used displays of four words (a fat-related word or a thin-related word, and three neutral words), which were presented for 8 seconds. Our primary dependent variable was the total fixation time for fat-related and thin-related words, defined as the sum of all fixation durations to these words during each 8-second presentation. By using both fat- and thin-related words, we were able to distinguish between a more specific attention bias for fat-related words and an avoidance bias for thin-related words versus a general attention bias for both types of weight-related words. As noted, distinguishing between these two manifestations of attentional bias has theoretical importance and could ultimately inform eating disorder prevention and treatment initiatives. Our results therefore have clinical implications.

The present study is the first to use eye-gaze tracking to measure attention to weight-related words over an extended interval, before and after thin model priming, which permitted a more detailed examination of how attention to weight-related words may change over time in women high and low in body dissatisfaction. Recall that Gao et al. [12] found that women high in body dissatisfaction exhibited heightened attention to both fat and thin body images compared to controls throughout the entire 15-second presentation time used in their study. This conclusion was based on a time course analysis, in which fixations to fat and thin body images were compared within each 3-second interval of the 15-second presentation. Given that analyses of the temporal patterns of attention could help clarify the mixed findings in the literature, we carried out a similar analysis to compare attention to weight-related words during the first 4 seconds versus the last 4 seconds of each 8-second presentation. The longer viewing time (relative to dot-probe studies) also allowed us to examine participants’ re-engagement of attention to weight-related words; that is, the likelihood that participants would return their gaze to fat- and thin-related words after the words were first examined. The frequency with which weight-related words are re-attended provides additional information on attentional biases related to body dissatisfaction because re-fixations of words reflect greater interest in their semantic content and higher perceived salience to the participant [35, 36].

Based on the findings of previous studies, we predicted that 1) there would be an interaction between baseline body dissatisfaction and thin model image priming on self-reported state body dissatisfaction: body-dissatisfied women would exhibit a larger increase in state body dissatisfaction than body-satisfied women after being exposed to thin model images, 2) body-dissatisfied women would exhibit biases in attentional engagement: they would have longer total fixation times for fat-related words than body-satisfied women, and shorter total fixation times for thin-related words (as reflected in attentional maintenance bias scores; Gao et al. [11]); 3) temporal analyses of attention would show that this pattern of attentional engagement would be present throughout the entire 8-second presentation (i.e., during the first 4 seconds and the last 4 seconds of each 8-second presentation); 4) body-dissatisfied women would be more likely to re-fixate on fat-related words and less likely to re-fixate on thin-related words than body-satisfied women, especially after the thin model priming (as reflected in their patterns of re-engagement of attention); 5) for body-dissatisfied women, the thin model priming would increase total fixation times for fat-related words and decrease total fixation times for thin-related words relative to the fixation times measured before the thin model prime.

## Materials and methods

The study involved three phases. First, we used an online survey to collect ratings for a large set of words and images to facilitate selection of an optimal set of stimuli to present to participants. Second, we screened prospective participants for eligibility to participate in the study via an online survey. Third, prospective participants who met eligibility criteria were invited to visit the laboratory to complete the eye-tracking phase of the study. Each phase is described below.

### Stimuli development

An online survey administered via Qualtrics (www.qualtrics.com) was used to collect ratings for words and thin model images. Participants provided informed consent before they began the survey. Each of the 64 female undergraduate students who completed the survey was presented with images of thin models, and with thin-related, fat-related, and neutral words. Students completed the survey in exchange for bonus credit in a psychology course. None of these students participated in the eye-tracking phase of the study. The students who completed the survey were a mixture of junior and senior students, and most were psychology majors.

To develop a large set of images of thin models, participants were asked to rate a randomly selected 50% of 40 images of thin female models; for each model they were asked to rate how closely the model depicted the thin-ideal standard portrayed in the mass media, using a Likert scale from 1 (“not at all”) to 5 (“very much”). The thin model images in the rating set included all 13 of the images used by Markis and McLennan [13], plus an additional 27 new images that were found on internet sites. All the models were in two-piece bra and underwear, and all the images had a neutral coloured background. Of the 40 images rated, we used the 25 images with the highest average ratings (*M* = 4.89, *SD* = 0.13) for the thin model priming procedure. By using more images than Markis and McLennan (25 images versus 13 images), we expected to increase the potency of the priming procedure.

To develop sets of thin-related, fat-related, and neutral words, participants were asked to rate a randomly selected 50% of 380 words on valence and category. The words rated included 18 thin-related words and 18 fat-related words that were validated and used in previous research [37], and an additional 22 thin-related and 22 fat-related synonyms. Thus, in total, there were 40 thin-related words, 40 fat-related words, and 300 neutral words to rate. Participants were asked to rate the valence of each word on a scale from –3 (“very negative”) to +3 (“very positive”). For the category rating participants were asked to categorize each word as either “thin,” “fat,” “neutral,” or “unsure”. For a word to be included in the final set of words, more than 70% of participants had to agree to the word’s category and less than 10% could be unsure of the word’s meaning. These criteria were used successfully in a previous study [37]. Of the words that met these criteria, the final thin-related, fat-related, and neutral word sets were created so that the sets were comparable in number of letters and normative frequency (calculated as the number of times a word appeared in print per one million words, as determined using SUBTLEXus, an index of American English; http://subtlexus.lexique.org/). As expected, the fat-related words were rated as more negatively valenced than both the thin-related words and the neutral words (see Table 1). There were 25 thin-related, 25 fat-related, and 150 neutral words in the final word set.

**Table 1:** Descriptive and inferential statistics for fat-related, thin-related, and neutral words.

	Fat-related Words	Thin-related Words	Neutral Words		
Word characteristic	*M (SD)*	*M (SD)*	*M (SD)*	*F*	*p*
**Number of letters**	6.04 (1.84)	7.00 (2.84)	6.52 (2.40)	1.00	.370
**Normative frequency**	11.28 (20.66)	13.88 (30.50)	14.01 (28.32)	.10	.901
**Rated valence**	–1.35 (0.55)	–0.38 (1.25)	–0.16 (1.05)	36.06	< .001

Normative frequency = frequency of the word in the English language per million words, determined using the SUBTLEXus database (http://subtlexus.lexique.org/); Rated valence = average rated valence on a scale from –3 (“very negative”) to +3 (“very positive”).

### Eligibility screening

To identify body-dissatisfied and body-satisfied women to participate in the eye-tracking phase of the study, a second online survey administered via Qualtrics was used to identify women with high and low body dissatisfaction. Female undergraduate students enrolled in psychology courses (*N* = 642) and 22 women from the campus community (recruited via posters placed on campus) completed the survey. The survey included the Body Shape Questionnaire (BSQ)[38], described below, as well as demographics questions, including age, education, and ethnicity. Total scores on the BSQ were calculated, with higher scores representing greater body dissatisfaction. Women were divided into top, middle, and bottom tertiles based on their BSQ scores. Although there are proposed classification scores for the BSQ that are intended to reflect various levels of concern with body shape and weight [38], we used a tertile split to create two large and widely separated groups of body-satisfied and body-dissatisfied women, of similar size. From the tertile split, there were 221 women scoring in the bottom tertile (BSQ scores ranging from 35–70), 222 in the middle tertile (BSQ scores ranging from 71–98), and 221 in the top tertile (BSQ scores ranging from 99–190). Only women who scored in the top (“body-dissatisfied”) or bottom tertile (“body-satisfied”) were invited to participate in the laboratory visit.

### Participants

Eligible women were invited via email for a laboratory visit to participate in the eye-tracking task. Only those whose first language was English were eligible to participate because the task involved reading many low-frequency words (e.g., *undernourished*, *heavyset*). Participation rates of those contacted via email were 24.7% from the top tertile and 25.6% from the bottom tertile. A total of 82 women were recruited for a laboratory visit. Two body-dissatisfied participants and two body-satisfied participants were not included in any analyses due to eye-tracking calibration issues and poor quality eye-tracking data, leaving a final sample of 78 women: 40 who scored in the top tertile (body-dissatisfied; BSQ scores ranged from 99–182) and 38 who scored in the bottom tertile (body-satisfied; BSQ scores ranged from 43–70). The mean age of the participants was 22.2 years (*SD* = 4.3), and 53.2% were Caucasian. Among those invited who did not participate in the eye-tracking phase, 53.8% were Caucasian, and the average age was 20.3 years (*SD* = 3.10). The final sample of 78 women consisted of 63 undergraduate students (who received bonus credit in a psychology course for participating) and 15 campus community participants (who received a $10 gift card). The study was approved by the University of Calgary Conjoint Faculties Research Ethics Board and all participants provided written informed consent.

### Materials and measures

#### Body Shape Questionnaire (BSQ)

The BSQ [38] is a 34-item self-report measure assessing body dissatisfaction. It assesses a respondent’s concern about body shape and the experience of “feeling fat” over the past four weeks (e.g., “Over the past four weeks, have you felt excessively large and rounded?”). Each item is rated on a 6-point Likert scale that ranges from “never” to “always”. Higher total scores reflect greater levels of body dissatisfaction. The BSQ has good reliability (test-retest reliability of 0.88; [39]) and good concurrent and discriminant validity [38].

#### Body Mass Index (BMI)

Participants’ heights and weights were self-reported, and their BMIs (kg/m^2^) were calculated from these data. The BMI is an estimate of adiposity [40]. High correlations (*r* > .90) have been found between self-reported and measured weight and height [41].

#### Body dissatisfaction Visual Analogue Scale (VAS)

Participants were asked to rate their current level of body dissatisfaction on a scale from 0 to 100, by drawing a mark on a 16 cm horizontal line with endpoints labelled “0 –Extremely *satisfied* with your body” and “100 –Extremely *dissatisfied* with your body.” The ratings were collected via paper and pencil. Body dissatisfaction scores were calculated as the percentage of the line to the right of 0 that a participant placed her mark.

#### Priming conditions

Following Markis and McLennan’s [13] procedure, we used a thin model priming task (presenting a set of 25 images of thin models validated in the stimuli development phase described above) and a control priming task (presenting a set of images of gender-neutral shoes, which included running shoes, hiking shoes, and sneakers). For the control priming task, the image set consisted of the 13 gender-neutral shoes used by Markis and McLennan and an additional 12 gender-neutral shoe images collected from the internet based on their similarity to the Markis and McLennan shoes. For both priming tasks a Microsoft PowerPoint 2010 slideshow was used to present the images, with each image presented for 10 seconds. There was a 3-second interval between the presentation of each image, during which participants were instructed to rate the image. For the thin model priming task, participants were asked to rate how closely each model matched their perception of the ideal female body as portrayed in the mass media, using a scale from 1 (“Not at all”) to 4 (“Very much”). These instructions disguised the fact that the purpose of viewing the images was to expose participants to images of thin models in an attempt to prime body dissatisfaction via social comparison. For the control priming task, participants were asked to rate how attractive each shoe was, using the same scale. The purpose of the control priming task was to engage participants in a similar experience as the thin model priming task, but without the possibility of priming any schema related to body image. Both priming tasks were approximately 6 minutes in duration.

#### Eye-tracking paradigm

Eye movements were recorded using an EyeLink 1000 eye-tracking system (SR Research Ltd., Ottawa, Ontario). The system uses infrared video-based tracking technology. Data were collected using a 1000 Hz sampling rate, with a temporal resolution of 2 milliseconds and an average gaze error of less than 0.5 degrees of visual angle. Words were presented on a 24-inch LCD monitor that was positioned approximately 60 cm away from the participant. Participants used a head rest to increase tracking accuracy. To ensure that participants were fixating in the centre of the display prior to the presentation of the words, at the start of each trial a fixation marker was presented in the centre of the display and the words were presented when the eye-tracking system determined the participant had fixated on the marker for 1 second.

For the eye-tracking task, participants were shown 75 sets of four words, each set presented for 8 seconds. There were 25 thin-neutral word sets (one thin-related word and three neutral words), 25 fat-neutral word sets (one fat-related word and three neutral words), and 25 neutral word sets (four neutral words). The neutral word sets served as filler trials to make the fat-related and thin-related words less conspicuous in the 75 sets of words presented. One word was presented in the top right, top left, bottom left, and bottom right of the display, and the location of the fat-related and thin-related words was randomly determined in the sets containing these types of words. Two blocks of word sets were presented, one set presented before the thin model image priming and one after; there were 37 word sets in one block and 38 word sets in the other (75 sets in total). The order of presentation of these blocks was counterbalanced across participants, and for each participant the word sets within a block were presented in a separate randomized order. Eye gaze was tracked and recorded throughout each 8-second presentation of a word set. The words in the thin-neutral and fat-neutral word sets were matched on number of letters and had similar printed frequencies and valence (the mean valence rating as determined in the stimuli development phase described above). Doing so ensured that the fat- and thin-related words were distinguishable from the neutral words in a set only in terms of their semantic content. The words in the neutral word sets were matched to the words in the other two sets on number of letters.

### Procedure

A within-subjects design was used to increase the ability to detect an effect of the thin model priming on attention, with attention to the fat- and thin-related words measured before and after exposure to the thin model prime. Participants completed the control priming task first, in which they viewed shoe images on a computer screen and rated them. Participants then completed the first eye-tracking task, which was administered on a different computer. For the eye-tracking assessment, they were told they would be shown sets of words and that the eye-tracking system would determine how their pupil dilation varied as a function of word familiarity (these instructions were intended to reduce demand characteristics that could affect participants’ attention to fat- and thin-related words). After eye-tracking data were collected for the first block of words, participants completed a body dissatisfaction VAS. Participants then returned to the priming task computer where they completed the thin model image priming task, rating each of the 25 thin model images. After this task was completed, eye-tracking data were collected for the second block of words. Finally, participants completed a second body dissatisfaction VAS. The second VAS was administered after the second eye-tracking block (instead of after the priming task) to ensure that attention was measured immediately after exposure to the prime (potentially increasing the sensitivity to a priming effect on attention). Participants were fully debriefed upon completion of the study.

### Data analysis

#### Data preparation

The fixation data were processed using the EyeLink Data Viewer analysis software (SR Research) to filter for blinks, missing data, and other recording artifacts (using the default settings). To be included in the analyses, a fixation had to be at least 100 ms in duration; adjacent sequential fixations less than 100 ms were merged into a single fixation. Following Gao et al. [11], attentional maintenance bias scores were created for each participant by calculating the time spent fixating on fat- and thin-related words (total fixation times summed across all trials) as a percentage of the total time spent fixating on all four words in each word set over the 8-second presentations. Higher scores therefore represent greater attentional maintenance (i.e., longer total fixation times). Re-engagement bias scores were computed for each participant by calculating the total number of times a fat- or thin-related word was the first word to be re-fixated as a percentage of the total number of trials that had at least one re-fixation. This analysis allowed us to examine whether there were biases in the likelihood that fat- or thin-related words were the first words to be re-attended, which would reflect the tendency of these words to re-engage participants’ attention.

#### Statistical analyses

Participants’ body dissatisfaction VAS scores were analyzed using a 2 (Group: body-satisfied, body-dissatisfied) x 2 (Prime Condition: pre-prime, post-prime) mixed-model analysis of variance (ANOVA). This analysis allowed us to determine if there was an effect of the thin model priming condition on self-reported state body dissatisfaction, and if this effect differed depending on pre-existing body dissatisfaction (hypothesis 1).

Attentional maintenance bias scores were analyzed using a 2 (Group: body-satisfied, body-dissatisfied) x 2 (Prime Condition: pre-prime, post-prime) x 2 (Word Type: fat-related, thin-related) mixed-model ANOVA, to determine if body-dissatisfied women would have longer total fixation times for fat-related words and shorter total fixation times for thin-related words than body-satisfied women (hypothesis 2). This analysis was also used to determine if the thin model priming would increase total fixation times for fat-related words and decrease total fixation times for thin-related words, particularly for body-dissatisfied women (hypothesis 5).

We also examined whether any difference between body-satisfied and body-dissatisfied women in their attention to weight-related words was maintained throughout the 8-second presentations (hypothesis 3). Recall that Gao et al. [12] found that body-dissatisfied women gazed longer at images of “fat” and “thin” bodies than controls throughout the entire 15-second image presentation used in their study (which was divided into five 3-second intervals for their analysis). In our analysis, we calculated attentional maintenance bias scores for the first 4 seconds of each presentation (the 0–4 second interval) and the last 4 seconds of each presentation (the 4–8 second interval). We analyzed only the data collected before participants were exposed to the thin model prime so that we could compare our results to those of Gao et al., who did not use a thin model priming procedure. For the analysis, attentional maintenance bias scores for weight-related words (the average of the fat- and thin-related words) were calculated separately for the 0–4 second and 4–8 second intervals and were analyzed using a 2 (Group: body-satisfied, body-dissatisfied) x 2 (Time Interval: 0–4, 4–8) mixed-model ANOVA.

Finally, re-engagement bias scores were analyzed using a 2 (Group: body-satisfied, body-dissatisfied) x 2 (Prime Condition: pre-prime, post-prime) x 2 (Word Type: fat-related, thin-related) mixed-model ANOVA to determine whether there were any differences between the body-satisfied and body-dissatisfied groups in their re-engagement of attention to the fat- or thin-related words before and after priming (hypothesis 4).

## Results

### Group characteristics

With respect to age, the body-satisfied women (*M =* 22.3 years, *SD =* 4.3) did not differ significantly from the body-dissatisfied women (*M* = 22.2, *SD* = 4.3), *t*(74) = 0.10, *p* = .917. The groups were also similar in ethnicity, Χ^2^(1, *N* = 77) = 1.10, *p* = .293, with 59.5% of the body-satisfied group and 47.5% of the body-dissatisfied group identifying as Caucasian. The body-satisfied group had lower BMIs (*M* = 20.9 kg/m^2^, *SD* = 3.1) than the body-dissatisfied group (*M* = 24.5, *SD* = 4.1), *t*(74) = 4.31, *p* < .001. As expected, BSQ scores differed between the groups, with body-dissatisfied women (*M* = 136.1, *SD* = 25.3) having significantly higher BSQ scores than body-satisfied women (*M* = 59.7, *SD* = 8.3), *t*(48) = 18.13, *p* < .001 (for this comparison the homogeneity of variance assumption of the standard *t*-test was violated and so the *t*-test probability was calculated assuming heterogeneity of variance).

We compared the undergraduate students (*n* = 63) and campus community participants (*n* = 15) at baseline on the variables of interest for hypotheses 1–5. The undergraduate students and campus community participants did not differ significantly on body dissatisfaction VAS scores (*M* = 39.4, *SD* = 24.0 vs. *M* = 51.4, *SD* = 22.8), *t*(76) = 1.76, *p* = .082 or on any of the attentional maintenance bias scores for fat-or thin-related words (all *p*s > .10) or attentional re-engagement bias scores for fat-or thin-related words (all *p*s > .10). These similarities justified combining the undergraduate students and community participants for all of the analyses.

### Body dissatisfaction VAS scores (hypothesis 1)

The interaction between Group and Prime Condition was significant, *F*(1, 76) = 8.22, *p* = .005, partial η^2^ = .10. Follow-up tests revealed that for body-dissatisfied women, body dissatisfaction VAS scores were higher post-prime (*M* = 64.0%, *SD* = 15.4) versus pre-prime (*M* = 58.3%, *SD* = 17.5), *t*(39) = 4.00, *p* < .001, whereas for body-satisfied women the difference between the post-prime (*M* = 24.9%, *SD* = 16.5) and pre-prime VAS scores (*M* = 24.2%, *SD* = 16.6) was not significant, *t*(37) = 0.63, *p* = .532. Thus, the thin model priming task was effective at increasing body dissatisfaction only for body-dissatisfied women.

### Attentional maintenance biases (hypothesis 2)

**Fig 1:**
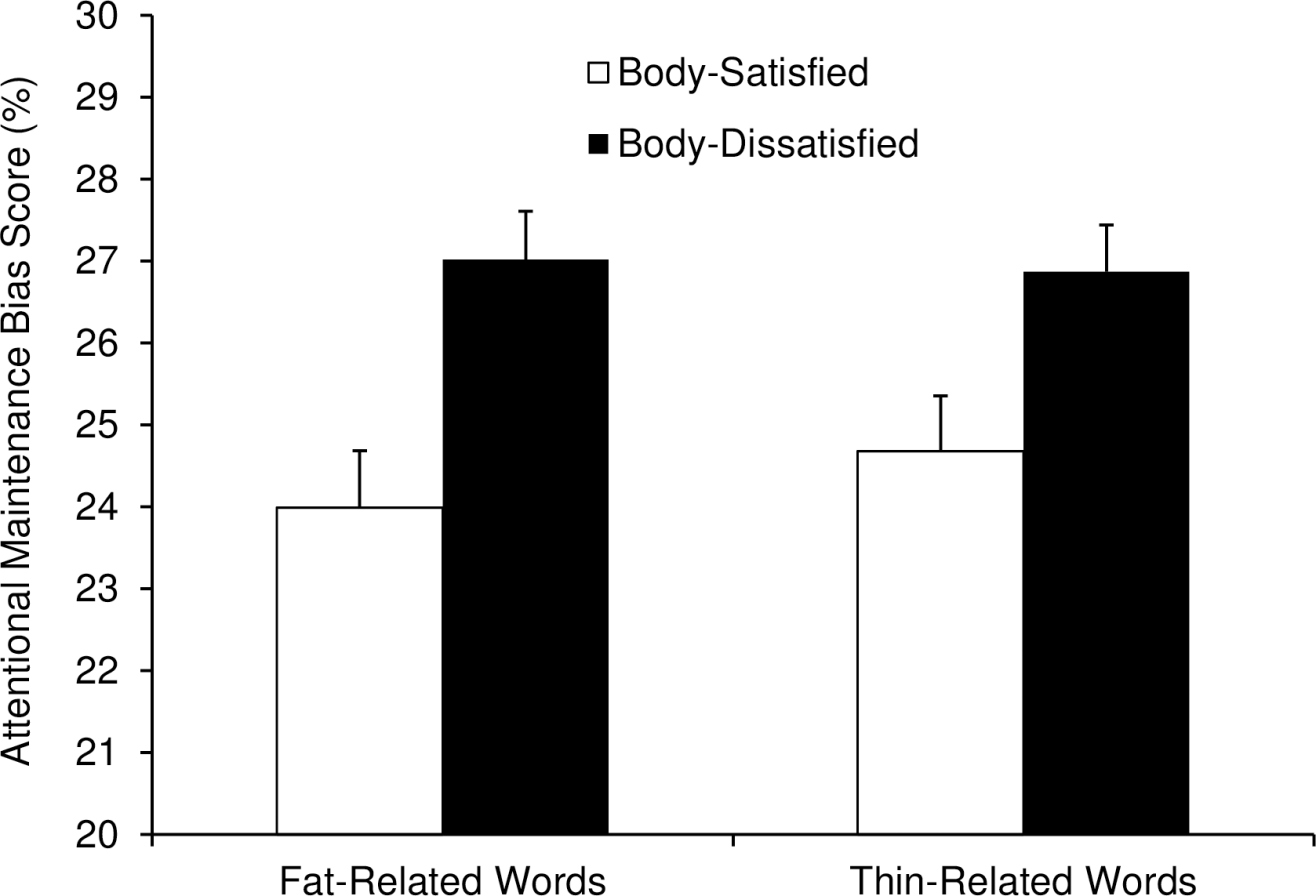
Attentional maintenance bias scores for fat-related versus thin-related words in body-satisfied and body-dissatisfied women, averaged across priming conditions. Attentional maintenance bias scores were calculated as the time spent fixating on fat- and thin-related words (total fixation times summed across all trials) as a percentage of the total time spent fixating on all four words in each word set over the 8-second presentations.

Table 2 lists the attentional maintenance bias scores for fat-and thin-related words for body-satisfied and body-dissatisfied women. Contrary to hypothesis 2, the interaction between Group and Word Type was not significant, *F*(1, 76) = 0.97, *p* = .327. That is, compared to body-satisfied women, body-dissatisfied women did not have longer fixation times for fat-related words and shorter fixation times for thin-related words. The analysis instead revealed a significant main effect of Group, with body-dissatisfied women fixating on both the fat- and thin-related words more than body-satisfied women (*M* = 27.0% vs. 24.3%), *F*(1, 76) = 10.98, *p* = .001, partial η^2^ = .13. Fig 1 shows the attentional maintenance scores as a function of Group and Word Type.

**Table 2:** Attentional bias scores for body-satisfied and body-dissatisfied women.

	Body-Satisfied (*N =* 38)	Body-Dissatisfied (*N =* 40)
	**Attentional maintenance bias scores (%)**	
**Pre-prime**	***M (SD)***	***M (SD)***
Fat-related words	23.40 (4.77)	26.39 (5.02)
Thin-related words	24.29 (3.86)	26.92 (4.69)
**Post-prime**		
Fat-related words	24.58 (4.75)	27.66 (5.80)
Thin-related words	25.08 (4.24)	26.82 (4.80)
	**Attention re-engagement bias scores (%)**	
**Pre-prime**	***M (SD)***	***M (SD)***
Fat-related words	8.74 (6.42)	10.06 (6.89)
Thin-related words	7.56 (6.22)	9.10 (4.39)
**Post-prime**		
Fat-related words	7.62 (5.57)	10.25 (6.43)
Thin-related words	7.16 (3.86)	8.07 (6.92)

Pre-prime = before exposure to the thin model images; Post-prime = after exposure to the thin model images; Attentional maintenance bias score = time spent fixating on fat- and thin-related words as a percentage of the total time spent fixating on all four words in each word set; Attention re-engagement bias score = total number of times a fat- or thin-related word was the first word to be re-fixated as a percentage of the total number of trials that had at least one re-fixation.

Note that the absence of an effect for Word Type for the body-dissatisfied women was not due to a lack of statistical power. For example, a post hoc power analysis indicated that with the sample of 40 women in the body-dissatisfied group, the power to detect a medium difference (*d* = 0.50) in attentional maintenance between fat- and thin-related words was 86.9% for a two-tailed test, using an alpha of 0.05 (calculated using G*Power 3.1; [42]).

### Temporal analysis of attentional engagement (hypothesis 3)

**Fig 2:**
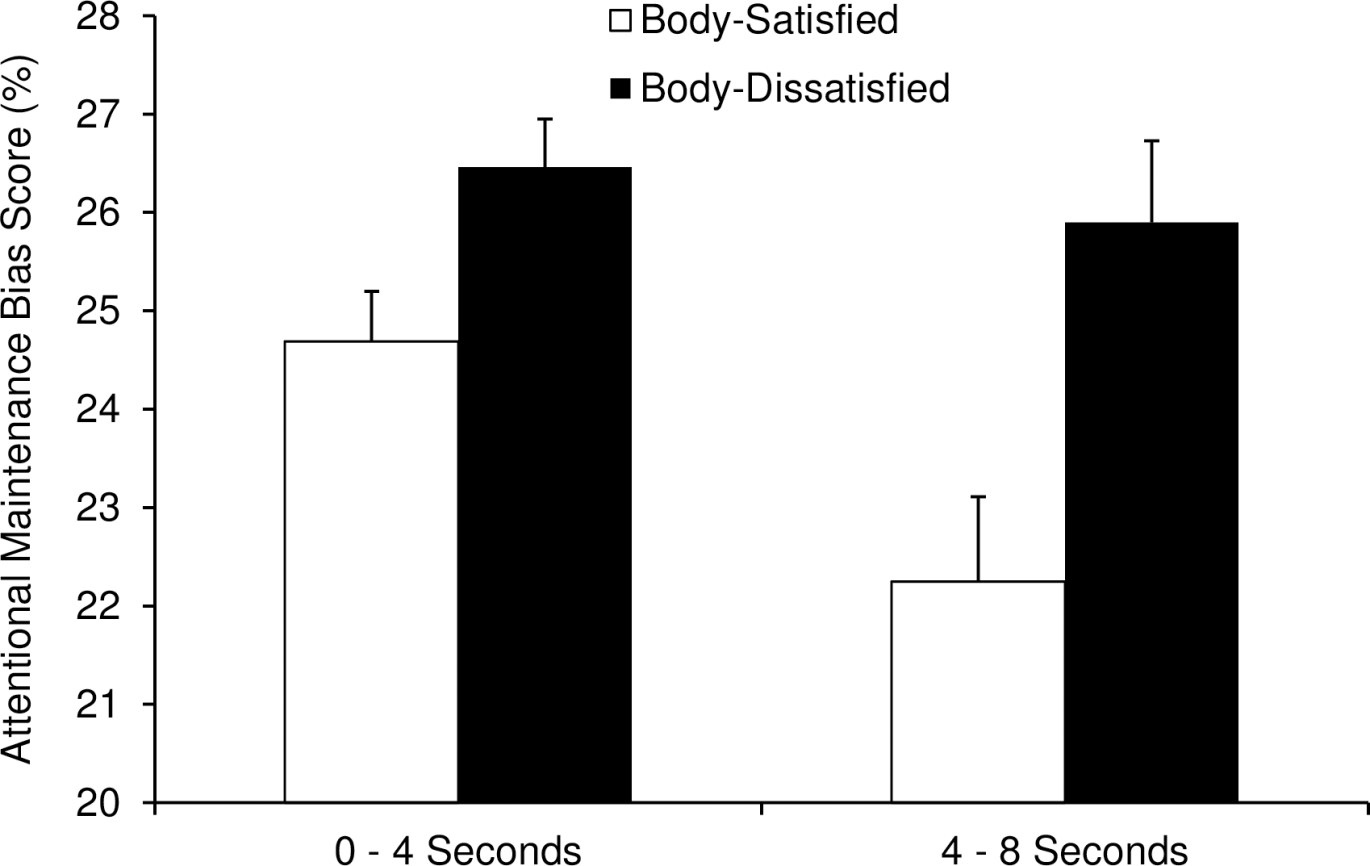
Attentional maintenance bias scores for weight-related words averaged across fat- and thin-related words, for each 4-second interval of the 8-second presentation, prior to the thin model prime. Attentional maintenance bias scores were calculated as the time spent fixating on fat- and thin-related words (total fixation times summed across all trials) as a percentage of the total time spent fixating on all four words in each word set.

This analysis revealed a significant main effect of Group, with body-dissatisfied women fixating on the weight-related words more than body-satisfied women (*M* = 26.2% vs. 23.5%), *F*(1, 76) = 11.14, *p* = .001, partial η^2^ = .13. There was also a significant main effect of Time Interval, with participants fixating on the weight-related words more during the 0–4 second interval (*M* = 25.6%) than during the 4–8 second interval (*M* = 24.1%), *F*(1, 76) = 7.58, *p* = .007, partial η^2^ = .09. Most important was the absence of an interaction between Group and Time Interval, *F*(1, 76) = 2.97, *p* = .089, which indicated that body-dissatisfied women attended to the weight-related words more than body-satisfied women during both intervals, as can be seen in Fig 2. Thus, consistent with the findings of Gao et al. [12] on attention to images of “fat” and “thin” bodies, we found that body-dissatisfied women’s heightened attention to weight-related words as compared to body-satisfied women was maintained throughout the 8-second presentations.

### Attention re-engagement biases (hypothesis 4)

Table 2 lists the attention re-engagement bias scores for fat-and thin-related words for body-satisfied and body-dissatisfied women. There was a main effect of Word Type, with participants’ first re-engagements more likely to be fat-related words (*M* = 9.2%) than thin-related words (*M* = 8.0%), *F*(1, 76) = 4.54, *p* = .036, partial η^2^ = .06. There was also a significant main effect of Group, as body-dissatisfied women had more frequent first re-engagements to both fat- and thin-related words than body-satisfied women (*M* = 9.4% versus 7.8%), *F*(1, 76) = 6.43, *p* = .013, partial η^2^ = .08. This result indicates that weight-related words were more likely to re-engage the attention of body-dissatisfied women compared to body-satisfied women. Contrary to hypothesis 4, the interaction between Group and Word Type was not significant, *F*(1, 76) = 0.44, *p* = .507. The Group x Word Type x Prime Condition interaction was also not significant, *F*(1, 76) = 0.51, *p* = .479, which indicated that the thin model priming did not affect body-dissatisfied and body-satisfied women’s first re-engagements to fat- and thin-related words differently.

### Attentional maintenance biases and thin model priming (hypothesis 5)

Contrary to hypothesis 5, the Group x Word Type x Prime Condition interaction was not significant, *F*(1, 76) = 0.49, *p* = .488. Body-dissatisfied women did not increase their attention to fat-related words after being exposed to the thin model images, nor did they decrease their attention to thin-related words. Instead, for both groups, the thin model priming had virtually no effect on attentional maintenance bias scores, *F*(1, 76) = 2.59, *p* = .111. In addition, the interaction between Word Type and Prime Condition was not significant, *F*(1, 76) = 1.55, *p* = .217, nor was the interaction between Group and Prime Condition, *F*(1, 76) = .97, *p* = .327, which indicated that there was no effect of the priming on attentional maintenance when averaged across Group or Word Type.

To increase the sensitivity of this analysis, we carried out a post hoc analysis to determine if the priming task affected the attention of body-dissatisfied women who exhibited a clear priming effect on self-reported body dissatisfaction. Meta-analyses [27, 28] have found that exposure to media portrayals of women produces changes in state body satisfaction pre-to-post prime, with average effect sizes that correspond to a change of approximately 10%. We therefore defined a priming effect as an increase of at least 10% pre-to-post prime on the body dissatisfaction VAS. To determine whether attentional maintenance biases varied as a function of Word Type and Prime Condition among this subset of body-dissatisfied women (*n* = 20), the attentional maintenance bias scores of these participants were analyzed using a 2 (Word Type: fat-related, thin-related) x 2 (Prime Condition: pre-prime, post-prime) within-subjects ANOVA. The interaction of Prime condition and Word Type was not significant, nor was the main effect of Prime Condition or the main effect of Word Type (all *p* values > .10). Thus, there was no evidence that the thin model prime affected attentional maintenance biases even among body-dissatisfied women who were demonstrably affected by the priming procedure.

## Discussion

The purpose of this study was to compare attentional biases to fat- and thin-related words in women with high and low body dissatisfaction, and to determine if the presentation of thin model images influenced these biases. For body-dissatisfied women, contrary to one interpretation of Vitousek and Hollon’s [16] cognitive model, our findings did not support the conclusion that fat-related information is schema-congruent and thin-related information is schema-incongruent. Instead, we found that body-dissatisfied women attended similarly to both fat- and thin-related words and they attended to both types of words more than body-satisfied women. Analyses of temporal changes in attention over the 8-second presentations, along with analyses of participants’ re-engagement with weight-related words, were consistent with this conclusion. Taken together, these results provide support for an interpretation of Vitousek and Hollon’s model that maintains that negative weight-related schemas produce biases in attention to any weight-related information. For body-dissatisfied women, both fat- and thin-related concepts appear to be incorporated into negative weight-related schemas, and the processing of any weight-related information is affected by these schemas. Our results are consistent with those of other investigators who have observed heightened attention to both fat- and thin-related words compared to neutral words in patients with anorexia nervosa [43] and to “fat” and “thin” body images in individuals with high levels of body dissatisfaction as compared to control individuals [12]. Our results also contribute to the literature suggesting that presentation of a thin model prime does not affect attention to weight-related information [9].

Our results differ somewhat from those of Gao et al. [11], who found that weight-dissatisfied women had shorter gaze durations for thin-related words than weight-satisfied women in a dot-probe task. As these researchers noted, however, a limitation of their study was that the word pairs were presented only for 1000 milliseconds; because the termination of participants’ first fixation occurred an average of 740 milliseconds after the word pair’s onset, there was little display time remaining to measure subsequent fixations. As a result, 1000 milliseconds may not have been long enough to fully evaluate the attention of weight-dissatisfied women. A major advantage of our methodology is the ability to examine attention to weight-related words over a much longer time interval. We found that body-dissatisfied women attended to fat- and thin-related words more than body-satisfied women throughout the 8-second presentations, showing sustained attentional maintenance biases throughout this interval. This finding is consistent with a different Gao et al. study that found that body-dissatisfied women attended to images of both “fat” and “thin” bodies more than controls throughout a 15-second presentation [12]. By using an 8-second presentation time, we were also able to examine the re-engagement of attention to fat- and thin-related words, and we found that body-dissatisfied women were more likely to re-engage attention to both fat- and thin-related words than body-satisfied women. This latter finding provides additional support that both types of weight-related words had greater salience for body-dissatisfied women.

With respect to the influence of thin model priming on attention, we found that priming did not affect body-dissatisfied or body-satisfied women’s attention to fat- or thin-related words. This outcome is consistent with a previous study that found that presentation of a thin model prime did not affect attentional biases in women with body image concerns [9]. Markis and McLennan’s [13] study therefore remains the only study to report an influence of brief exposure to thin models on attention to body-related information in body-dissatisfied women. Note that in the present study, there was an effect of the prime on self-reported body dissatisfaction for body-dissatisfied women, as they reported greater state body dissatisfaction after viewing thin model images, whereas the priming procedure had no discernable effect on body-satisfied women. Thus, while one could argue that the absence of a priming effect on body-satisfied women’s attention was due to the ineffectiveness of the thin model priming procedure for these women, this reasoning would not apply for the body-dissatisfied women.

Like other researchers, we found that brief exposure to images of thin models can affect explicit thoughts of state body dissatisfaction in women with high levels of body dissatisfaction [27, 28]. However, it is important to note that not all researchers have observed this phenomenon. For example, Loeber et al. [44] found that 10 minutes’ exposure to thin models in fashion magazines in a simulated waiting room, with no explicit instructions to direct women’s attention to the images, did not trigger self-reported body dissatisfaction. Thus, the duration and nature of the priming procedure (including the specific instructions provided to participants, such as whether to engage in social comparisons when viewing thin models) may be critical to producing a substantial priming effect. Casually viewing such images may not be potent enough to heighten attentional biases in women with high levels of body dissatisfaction. That is, it is possible that brief experimental exposure to images of thin models, while having a measurable effect on self-reported body dissatisfaction, is not potent enough to activate an individual’s maladaptive body image schema to the extent that attention to weight-related words is affected.

### Limitations

Although this study had several methodological strengths, there were several limitations that should be taken into account when interpreting our findings. First, paradigms that assess attentional biases for disorder-salient words compared to either neutral words or control participants have been criticized as lacking clinical relevance [23]. This criticism deserves consideration and further study. Second, the use of words as stimuli may have limited our ability to detect group differences in attentional biases; it is possible that body-dissatisfied women would selectively attend more to “fat” images or videos of people (and less to “thin” images or videos) compared to body-satisfied women (although note that this is not what Gao et al. [12] found when they used images of “fat” and “thin” women’s bodies in their study). Third, because our study included only body-satisfied and body-dissatisfied women from a campus community, our findings cannot be generalized to clinical samples. Along these lines, the low participation rate of prospective participants that had been screened for the study may have also limited the generalizability of the results. Finally, as noted, brief exposure to images of thin models, while having a measurable effect on self-reported body dissatisfaction, may not have the ability to activate an individual’s maladaptive body image schema and influence attention to weight-related words. A longer prime duration, or a different priming procedure (e.g., self-reflection tasks; instructions to engage in social comparisons; audiovisual presentations) may be more effective. An important methodological issue for future research will be to identify the requirements of a potent priming procedure for body-dissatisfied women.

### Implications and directions for future research

The finding that body-dissatisfied women attend to both fat- and thin-related words more than body-satisfied women has implications for body dissatisfaction maintenance, treatment of body dissatisfaction, and eating disorder prevention among young women with body dissatisfaction. Exposure to thin-ideal media contributes to body dissatisfaction [45], and the present results add to the literature indicating that heightened body dissatisfaction can lead to attentional biases that can maintain maladaptive body image schemas. The reciprocal nature of these etiological and maintenance factors reinforces the importance of treatment and prevention. Whereas additional research is needed to determine whether the present findings generalize to clinical samples of individuals with an eating disorder, note that Vitousek and Hollon’s [16] model was originally developed to describe cognitive biases in individuals with an eating disorder (e.g., anorexia nervosa, and bulimia nervosa). Given that the present sample was screened for body dissatisfaction, an eating disorders risk factor [6, 7], it is possible that the general weight-related attentional bias observed in this sample partially extends to clinical samples.

Understanding how fat- and thin-related information is processed by body-dissatisfied women who are at risk for developing an eating disorder may inform cognitive-behavioural approaches to prevention and treatment [46]. For example, given our findings, both fat- and thin-related cognitive biases should be considered as potential mechanisms influencing the development and maintenance of maladaptive schemas. Therefore, attentional bias modification programs (e.g., [25]) designed to reduce attentional biases in those at risk of developing an eating disorder should target both fat- and thin-related content to alter maladaptive schemas related to body dissatisfaction most effectively. As the attentional bias we have observed could generalize to a broader category of words (e.g., all body-related words), this possibility should be explored in future research. In addition, future research should explore whether our findings based upon attention to words extend to images. This is an important question to explore, as women are routinely exposed to a multitude of weight-related images in western society’s high density visual media environment, and this research could increase generalizability of results. Our study also highlights the usefulness of additional eye-tracking research to more comprehensively understand the nature of negative weight-related schemas across diverse samples, including both clinical and non-clinical samples of women.

Another possibility for future studies is to assess the automatic thoughts individuals experience while they engage in attention tasks related to body image, to better understand the mechanisms affecting biased information processing in body-dissatisfied individuals. For example, if a young woman engages in upward social comparison thoughts when viewing thin-related information and downward social comparison thoughts when viewing fat-related information [18, 47], then targeting these cognitions using cognitive reframing strategies from cognitive behavioural treatment techniques [48] may help refine prevention and treatment efforts. Such a strategy may have the potential to reduce cognitive biases related to maladaptive weight-related schemas. Thus, as Vitousek and Hollon [32, 16] discussed, there is an urgent, practical need for a better understanding of the unique cognitive processes associated with eating pathology so that treatment can target schema-related biases.

## Conclusion

The present study examined attention to fat- and thin-related words in body-dissatisfied and body-satisfied women before and after the women were exposed to a thin model priming procedure. Although previous empirical tests of cognitive theories of body dissatisfaction have yielded conflicting results, the present study, the first to use eye tracking to assess both the engagement and re-engagement of attention to fat- and thin-related words, provided compelling evidence for the existence of heightened attention to both fat- and thin-related words. We found that body-dissatisfied women attended to both fat- and thin-related words more than body-satisfied women throughout the 8-second presentations. These results support an interpretation of Vitousek and Hollon’s [14] cognitive model that the processing of all weight-related information is affected by negative weight schemas. An additional contribution is the finding that the presentation of thin model images did not alter women’s attention to fat- and thin-related words, even though the priming affected body-dissatisfied women’s self-reported body satisfaction. Our study has identified several promising directions for future research.
